# Adsorption of Dyes, Pharmaceuticals, and Humic Acids onto Chitosan Biomaterials Doped with Activated Carbon: Colloidal Approaches and Interaction Explanations

**DOI:** 10.1021/acs.langmuir.5c05055

**Published:** 2025-12-01

**Authors:** Konstantinos N. Maroulas, Athanasia K. Tolkou, Dimitrios Theologis, Margaritis Kostoglou, Ioannis A. Katsoyiannis, George Z. Kyzas

**Affiliations:** † Hephaestus Laboratory, School of Chemistry, Faculty of Sciences, 37791 Democritus University of Thrace, GR-65404 Kavala, Greece; ‡ Laboratory of Chemical and Environmental Technology, Department of Chemistry, 37782 Aristotle University of Thessaloniki, GR-54124 Thessaloniki, Greece

## Abstract

Various pollutants affect the hazardousness of wastewater, with organic pollutants playing a main role. Thus, among others, wastewater contains natural organic matter (NOM), pharmaceutical compounds, and various toxic and nonbiodegradable dyes. Removing these hazardous compounds from water is essential since they are harmful to both human health and water quality. Therefore, in this study, we evaluated the removal of humic acids (HA), Diclofenac (DCF), and Reactive Red 120 (RR120) by using chitosan/activated carbon and chitosan/activated carbon/curcumin derivatives as adsorbents. Several derivatives with different composition ratios, such as CS/AC@1:1, CS/AC@1:2, and CS/AC@2:1, were initially synthesized and examined. Furthermore, the addition of curcumin (Cur), which is a natural polyphenolic substance, to the CS/AC@ derivative, i.e., CS/AC@Cur5% and CS/AC@Cur10%, was also examined, in order to scrutinize the possibility of additional enhancement of organic contaminants removal. It was found that at pH 2.0, more than 93% of HA was removed by applying CS/AC2:1 (109 mg/g). In the case of DCF at pH 6.0, 97% was removed by CS/AC@Cur5% (148 mg/g), but CS/AC@Cur10% (112 mg/g) was optimum for RR120 removal (94%) at pH 3.0 and 293 K. The adsorption data were more consistent with the Langmuir isotherm model, whereas the adsorption kinetics followed the pseudo-second-order model in all cases. Thermodynamic analysis confirmed that the adsorption was endothermic and spontaneous for all of the organic pollutants. Adsorption–desorption experiments confirmed that these adsorbents can be used effectively for up to six cycles of regeneration.

## Graphical abstract

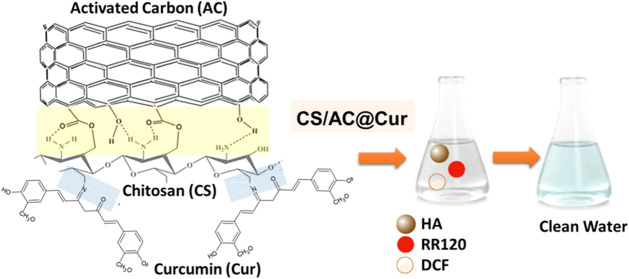

## Introduction

Organic pollutants, such as dyes, humic substances, various phenolic compounds, pesticides, pharmaceuticals, etc., are hazardous pollutants in wastewater, as they can produce toxic chemicals during disinfection.^1^ Natural organic matter (NOM) is one of the major contaminants affecting the quality of water and the aquatic environment.^2^ It has been noted that NOM, which is created by metabolic processes in water, results in a variety of issues, including an unpleasant taste and color, the development of disinfection byproducts (DBP), and a decrease in the quantity of dissolved oxygen in water. Humic acid (HA) and fulvic acid (FA) are the main components of NOM.^3^ Humic substances make up the largest portion of NOM. The International Agency for Research on Cancer (IARC) has classified HA as a possibly carcinogenic substance. Other health consequences include central nervous system issues, reproductive abnormalities, anemia, Blackfoot disease, and cytotoxic effects.^4^ While HA substances have a negative impact on human health and water quality, it is crucial to remove them from water.

The textile industry places a significant chemical burden on the environment, primarily by releasing dyes and associated coloring chemicals in its effluents. Because most dyes are synthetic, their presence and accumulation pose a serious hazard to the environment.^5^ Furthermore, the intense color of dyes reduces sunlight penetration into water bodies, halting photosynthesis in aquatic systems.^6^ Azo dyes are unique dyes with an additional azo group (−N=N−), which stabilizes the dye molecules toward heat, light, and aerobic digestion. The consumption of azo dyes through food or drink can result in acute vomiting, allergic reactions, cyanosis, and genetic mutations. Reactive Red 120 (RR120) is one of the dyes frequently used in textile industries and is a possible concern to the aquatic system because of its poor biodegradability.^7^ Additionally, due to the inclusion of amines and related groups, it is highly poisonous and carcinogenic, posing substantial risks to all living organisms.^8^

Furthermore, the ever-increasing use of drugs and their subsequent release into the environment pose a threat to life. Diclofenac (DCF), a pharmaceutical product belonging to the class of nonsteroidal anti-inflammatory drugs, is a widely used painkiller for both humans and animals in general.^9^ Due to the prevalence of lifestyle-related diseases, such as arthritis in particular, but also heart disease, its use and the risks it poses have increased. Therefore, although DCF has many pharmaceutical advantages, it is frequently detected in various environmental sources, and its persistence has harmful effects on aquatic ecosystems.^10^ Therefore, there is an urgent need for effective management in wastewater treatment plants.

This study investigates the simultaneous removal of dyes, pharmaceuticals, and humic acids, which are three classes of contaminants that represent major challenges in contemporary wastewater treatment. Synthetic dyes originating from textile effluents,^11^ pharmaceuticals derived from municipal and hospital discharges,^12^ and naturally occurring humic substances frequently coexist in aquatic environments,^13^ albeit at different concentrations.

Numerous methods, including adsorption, membranes, enhanced oxidation, and photocatalytic oxidation, have been investigated for the removal of organic pollutants from water.^14–18^ Depending mostly on the kind of adsorbent chosen for the target adsorbate, adsorption has been used over the past ten years as an effective and economical method to remove a variety of organic and inorganic contaminants.^4,19–21^ According to published research, modified adsorbents, composite materials, and nanomaterials have demonstrated several favorable results for the removal of NOM,^4^ dyes,^22^ and pharmaceuticals^23^ from water. Because of its large surface area and well-developed porous structure, which provides an efficient adsorption capacity, activated carbon^24^ has been chosen as a perfect adsorbent. In addition, chitosan, an alternative biopolymer-based adsorbent, has recently attracted a lot of interest.^21,25^ Chitosan’s amine (−NH_2_) and hydroxyl (−OH) functional groups make it a potentially effective adsorbent and enhance its modifiability and possibility for cross-linking.^19,26,27^ However, the use of raw chitosan in batch or column modes is limited due to many significant issues with the adsorption process, including (i) poor mechanical properties, (ii) facile dissolution in acidic solutions, (iii) low specific gravity, and (iv) large swelling ratios.^28,29^ The practical use of typical chitosan-based biosorbents is also limited since they are challenging to separate and recover without the use of high-speed centrifugation or filtering. Due to the aforementioned factors, it is important and required to investigate environmentally friendly biosorbents made of raw chitosan that have superior separation properties and better adsorption capacities.^30^ To overcome this, a chitosan framework containing activated carbon was used.^31^ Chitosan is selected as an encapsulating material because of its affordability, mass availability, nontoxicity, and biodegradability.^28,32,33^

In addition, curcumin has been extensively investigated as a natural adsorbent for water purification.^34,35^ Adsorbents based on curcumin have drawn extensive attention and have been successfully used to purify water. Curcumin is often used as an anti-infection, immune-regulating, anti-inflammatory, and anticancer medication due to its strong pharmacological action.^36^ It is a natural pigment derived from turmeric and has shown great performance as an adsorbent for the removal of organic contaminants from wastewater.^37–39^ It is easily obtainable from turmeric, making it a sustainable and cost-effective choice.^40^ Unfortunately, because the stability and solubility of curcumin are deficient, in order to increase them, many techniques have been proposed, including graft modification.^34,41,42^

Chitosan-based composites have been widely used for the adsorption of organic pollutants. Dehghani et al. blended chitosan with zeolite and focused on the RR120 removal. Using Response Surface Methodology, they found that the optimal conditions were pH 5, dosage 2 g/L, and contact time 138.75 min; however, the maximum adsorption capacity was only 19.14 mg/g.^43^ In another study, glutaraldehyde cross-linked chitosan was modified with TiO_2_, resulting in a 103.1 mg/g adsorption capacity due to the presence of multiple adsorption mechanisms like H-bonding, electrostatic, and *n*–π interactions.^44^ Chitosan has also been utilized for the removal of HA. In a study by Zhang et al., a cellulose acetate/chitosan fiber composite managed to eliminate up to 184.72 mg/g of HA within 45 min.^45^ Regarding DCF, there are many examples where chitosan/activated carbon materials have been employed. In particular, Kumar et al. found that by using 1.5 g/L of commercial activated carbon/chitosan beads and adjusting the pH to 6, they managed to remove 99.29 m/g of the pollutant.^45^ Dago-Serry et al. used the same composite, but the activated carbon was derived from coconut shells.^31^ In this case, 165.33 mg/g DCF was removed at 323 K and pH 2.

In this study, the removal of hazardous organic contaminants, such as HA, RR120 dye, and DCF, by using chitosan/activated carbon or chitosan/activated carbon/curcumin derivatives, was evaluated. Glutaraldehyde was selected as a cross-linking agent, which results in strong chemical cross-linking. Knowing that it can be toxic at concentrations higher than its maximum allowed levels, extensive washing of the adsorbents using a Soxhlet apparatus was performed. Previous studies by our group regarding the cytotoxicity assessment of chitosan-based adsorbents, where glutaraldehyde was utilized, showed that the toxicity was negligible, rendering the adsorbents eco-friendly.^46^ The goal is to create an efficient adsorbent material that uses minimal additional substances to remove organic contaminants completely. To the best of our knowledge, no studies have investigated the use of chitosan/activated carbon/curcumin materials for the removal of all the aforementioned pollutants. The major parameters included the dosage of the adsorbent, initial concentration of the pollutant, pH, and contact time. The adsorption process is interpreted using various isotherms and kinetic models, and several factors, including thermodynamics, are investigated to better comprehend and assess the adsorption process. Complete material characterization is performed pre- and postadsorption on the optimal materials.

## Materials and Methods

All reagents used were of analytical grade. 0.01 g of humic acid (Sigma-Aldrich, Merck KGaA, Darmstadt, Germany) was dissolved in 50 mg/L distilled water to create stock humic acid aqueous solutions. In addition, 2 mL of 0.5 M NaOH was added to dissolve the humic acid.^47^ The stock solution was then diluted to provide the necessary concentrations of humic acid. Stock solutions of the RR120 dye (aqueous solution) and DCF in absolute methanol were then prepared. Chitosan (310–375 kDa, DDA > 75%) was purchased from Sigma-Aldrich (Merck KGaA, Darmstadt, Germany), as well as glutaraldehyde (50 wt % in H_2_O), Reactive Red 120 azo dye (Dye content ≥50%), and Diclofenac sodium (99%). Additionally, acetic acid (≥99%) (Fisher Chemicals, Hampton, New Hampshire) and sodium tripolyphosphate (TPP, Na_5_O_10_P_3_, molar mass 367 g/mol) (Alfa Aesar, Thermo Fisher Scientific Inc., Massachusetts) were used as cross-linkers. Curcumin (>70%) was purchased from Glentham Life Sciences (U.K.), and activated carbon was sourced from Sigma-Aldrich (Merck KGaA, Darmstadt, Germany). Finally, when necessary, HCl 37% (Panreac, AppliChem, Barcelona, Spain), or NaOH ACS reagent, ≥97.0%, pellets (Sigma-Aldrich, Merck KGaA, Darmstadt, Germany) were used to adjust the pH of the solution.

For the synthesis of chitosan/activated carbon derivatives (CS/AC@), 1% w/v chitosan was prepared by dissolving chitosan powder in aqueous acetic acid solution (2% v/v), based on a previous study.^31^ Then, activated carbon powder was added in different ratios; hence, three different CS/AC solutions were obtained with ratios of 1:1, 1:2, and 2:1, abbreviated hereafter as CS/AC@1:1, CS/AC@1:2, and CS/AC@2:1. The mixtures were subjected to ultrasound treatment for 1 h in order to disperse the AC. Glutaraldehyde (GLA) (50% in H_2_O) was then added to each derivative, and subsequently stirred again until the solutions turned to gel phase. The sol–gels that were formed were placed in a freezer for 24 h. They were freeze-dried at −104 °C for 48 h to produce the aerogels.^48–50^ Finally, the aerogels were ground into powder and purified with a water/methanol mixture in a Soxhlet apparatus for 24 h.

Regarding the synthesis of chitosan/activated carbon@curcumin derivatives, and based on preliminary experiments, the CS/AC@2:1 ratio was found to be the best. The latter was modified by the addition of curcumin, dissolved at concentrations of 5 and 10% by weight relative to chitosan, using acetone as the solvent under vigorous stirring and heating at 50 °C until acetone evaporated. The mixtures, CS/AC@Cur5% and CS/AC@Cur10%, were sonicated for 1 h to achieve dispersion of both AC and curcumin. Then, the GLA solution was poured, and the formed sol–gels were stored in the freezer for 24 h. The sol–gels were then lyophilized at −104 °C for 48 h, and finally, the aerogels were obtained, which were then ground into a fine powder and purified in a Soxhlet apparatus with a water/methanol mixture for 24 h. Thus, Table 1 summarizes all of the synthesized adsorbents used in this study.

**Table 1:** Composition of the Synthesized Adsorbents

A/A	adsorbent	constituents
1	CS/AC@1:1	chitosan and activated carbon (ratio 1:1)
2	CS/AC@1:2	chitosan and activated carbon (ratio 1:2)
3	CS/AC@2:1	chitosan and activated carbon (ratio 2:1)
4	CS/AC@Cur_5%	chitosan, activated carbon (ratio 2:1), and curcumin 5%
5	CS/AC@Cur_10%	chitosan, activated carbon (ratio 2:1), and curcumin 10%

To determine the residual concentrations of the pollutants (after adsorption) analytically, water samples were obtained from the supernatant of each sample, filtered through a filter (0.45 μm), and stored for further analysis. The relative residual concentration was determined by fitting the absorbance obtained by a ultraviolet–visible (UV–vis) spectrophotometer (WTW Spectroflex 6100, Weilheim, Germany) at λ_max_ 254 nm for humic acids, λ_max_ 512 nm for RR120, and λ_max_ 276 nm for DCF,^51^ to the calculated calibration curve (RR120: *R* ^2^ = 0.9979; DCF: *R* ^2^ = 0.9953; HA: *R* ^2^ = 0.9987).

To carry out the batch adsorption experiments, a number of tests were conducted to determine the effectiveness of chitosan/activated carbon or chitosan/activated carbon/curcumin derivatives as adsorbents for the removal of organic pollutants by adding an appropriate dosage of adsorbents to 10 mL of HA, DCF, or RR120 solutions. Throughout the tests, the mixture was stirred at a constant temperature. Various experimental factors were adjusted individually in each experiment while ensuring that all other parameters remained constant. These factors included pH levels (ranging from 2.0 to 11.0), initial pollutant concentrations (ranging from 2 to 450 mg/L), adsorbent doses (ranging from 0.1 to 1.0 g/L, according to preliminary experiments), and contact time (ranging from 5 to 1440 min (24 h)). After adsorption, the filtrate was used for subsequent analyses.

The percentage removal (*R*, %) was determined from the following equation (eq 1)­

1 $$R\left(\%\right)=\left(\frac{C_{0}-C_{f}}{C_{0}}\right)\times 100\%$$

where *C*_0_ is the initial pollutant concentration (mg/L), and *C*_f_ is the final pollutant concentration after treatment (mg/L),

The adsorption capacity of the adsorbent (*Q*_e_) (mg/g) was calculated from the following equation (eq 2):

2 $$Q_{e}=\frac{\left(C_{0}-C_{e}\right)\times V}{m}$$

where *C*_e_ indicates the pollutant concentration (mg/L) at equilibrium, *V* (L) is the volume of the solution, and *m* (g) is the mass of the adsorbent.

For the isothermal studies, 10 mL of HA, DCF, or RR120 solution with concentrations ranging from 2 to 60 mg/L for HA and 5–450 mg/L for DCF and RR120 was mixed with a constant amount of adsorbent in 15 mL Falcon tubes. Langmuir^52^ and Freundlich^53^ isotherm models were used to assess the results from these experiments. The Langmuir model is represented by eq 3.

3 $$Q_{e}=\frac{Q_{m}K_{L}C_{e}}{1+K_{L}C_{e}}$$

where *Q*_e_ represents the concentration of the adsorbate in the solid phase to the concentration in the liquid phase at equilibrium (mg/g), *Q*_m_ represents the maximum adsorption capacity, that is, the theoretical monolayer capacity (mg/g), and *K*_L_ represents the energy associated with the adsorption of the organic pollutants (L/mg).

According to Langmuir theory, there is no interaction between adsorbed molecules throughout the adsorption process since the adsorbate forms a single layer on the surface of the adsorbent. Furthermore, the theory assumes the limited adsorption capacity (*Q*_m_) of the adsorbent, which indicates the maximum amount of adsorbed substance that the adsorbent surface may absorb when equilibrium conditions are achieved.

The relationship between the equilibrium concentrations of HA, DCF, and RR120 (in mg/L) and the adsorbent’s adsorption capacity, *Q*_e_ (in mg/g) is described by the Freundlich model. eq 4 is the mathematical expression for this relationship.

4 $$Q_{e}=K_{F}C_{e}^{1/n}$$

where *K*_F_ ((mg/g)·(L/mg)^1/*n*^) is a constant related to the adsorption capacity, and 1*/n* is a constant related to the surface heterogeneity or adsorption intensity.

The contact time has an impact on the adsorbents’ ability to remove humic acid as well. At optimal pH values and constant temperatures, kinetic experiments were performed at different contact times. There are two major categories of models used to describe adsorption kinetics. The first category includes empirical models. Typically, these models are by far the most frequently used in the literature’s adsorption studies. Typical empirical models are the pseudo-first-order (PFO),^54^ pseudo-second-order (PSO),^55^ and Elovich models. This study examined the PFO and PSO kinetics of the HA adsorption. The resulting kinetic adsorption values were then examined in order to estimate the sorption rates and identify the appropriate rate expressions, characterizing the possible reaction mechanism. The pseudo-first-order and pseudo-second-order models used for the data analysis are presented in eqs 5 and 6, respectively:

5 $$Q_{t}=Q_{e}\left(1-e^{-k_{1}t}\right)$$

6 $$Q_{t}=\frac{k_{2}Q_{e}^{2}t}{1+k_{2}Q_{e}t}$$

where *Q*_t_ and *Q*_e_ are the amounts of HA adsorbed (mg/g) at time *t* (min) and at equilibrium, respectively, *k*_1_ is the pseudo-first-order rate constant (1/min), *k*_2_ is the relative pseudo-second-order rate constant (g/mg min), and *t* is the contact time (min).

In the majority of cases, the pseudo-second-order model appears to describe the adsorption kinetic data very accurately.^55^ Despite the pathologies associated with the fitting procedure leading to the wrong impression of perfect fitting, as shown by Simonin^56^ in the past and by Kostoglou and Karapantsios^57^ more recently, the pseudo-second order is still used in the relative literature. Therefore, for the sake of completeness, it is also demonstrated here. A basic characteristic of the empirical models is that they do not take into account the mass balance of the solute, as adsorption proceeds and the solute concentration decreases, thereby reducing the driving force for adsorption. Another characteristic of empirical models is the lack of a physical sense of their parameters. In this way, they cannot be used for adsorption conditions different from those prevailing in their derivation experiments.

The above characteristics do not fit phenomenological models, which are the second category of models. This means that they take into account solute mass balance, and their parameters have a direct physical meaning. A particular phenomenological approach to the adsorption kinetics problem is the one based on chemical reaction engineering principles.^58^ It considers instantaneous local adsorption–desorption of the solute on the walls of the adsorbent particle pores. The solute generally moves toward the interior of the adsorbed particle in two ways: pore diffusion through the liquid phase and surface diffusion on the pore walls. The generalized model consists of a complicated set of transient partial differential equations and an algebraic equation. Typically, in practice, in the context of analyzing experimental data, the mathematical problem is simplified by considering the surface diffusion as dominating the intraparticle solute transport and by using an order reduction mathematical procedure (i.e., linear driving force formula), which transforms the partial differential equation to an ordinary one.^59^ According to this model, the evolution of the adsorbed quantity is described by the following equation (eq 7):

7 $$\frac{dQ_{t}}{dt}=K\left(f\left(C\right)-Q_{t}\right)$$

where *f­(C)* is the adsorbed quantity in equilibrium with the instantaneous concentration *C* (mg/L) of the solute in the bulk. The concentration at each moment can be determined using the following solute mass balance (eq 8):

8 $$C=C_{0}-Q_{t}\left(\frac{m}{V}\right)$$

where *C*_0_ is the initial solute concentration.

The function *f­(C)* is the Langmuir isotherm corresponding to each adsorbed material. Equation 7 states that the adsorption rate is proportional to the deviation from equilibrium. A characteristic of this modeling approach is that the equilibrium isotherm is incorporated into the kinetic model. The constant *K* is related to the intraparticle surface diffusivity and in the general case is a function of the loading *Q*_t_. The following functional (eq 9) form of this relation is usually considered:

9 $$K\left(Q_{t}\right)=K_{o}/\left(1+k_{h}Q_{t}^{y}\right)$$

where *K*_0_ (1/min), *k*_h_ (g/mg)^3^, and *y* are the model parameters.

This form implies the inhibition of diffusion as loading increases, and its use is necessary to reproduce the apparent two-stage reduction of the adsorption rate observed in practice. The system of equations (eqs 7–9) combined with the Langmuir isotherm is solved numerically and then fitted to the experimental data.

Another important factor in the adsorption process is temperature; the relative effect on the organic pollutant adsorption was determined at different temperatures (293, 303, 313, and 323 K) when the optimum dosage and pH value were applied for 120 min as the contact time. The nature of the adsorption process is cautiously defined by thermodynamic evaluation and searching for changes in enthalpy (Δ*H* ^0^, kJ/mol), entropy (Δ*S* ^0^, kJ/mol·K), and Gibbs free energy (Δ*G* ^0^, kJ/mol). Therefore, eqs 10–13 were used to calculate the thermodynamic parameters at four different temperatures (293, 303, 313, and 323 K).

10 $$K_{c}=\frac{C_{s}}{C_{e}}$$

11 $${\Delta G}^{0}={-\mathrm{RT}\;\ln(K}_{c})$$

12 $${{\Delta G}^{0}=\Delta H}^{0}-T{\Delta S}^{0}$$

13 $${\ln(K}_{c})=\left(-\frac{{\Delta H}^{0}}{\mathrm{RT}}\right)+\frac{{\Delta S}^{0}}{R}$$

where *K*_c_ is the thermodynamic constant, *R* is the universal gas constant (8.314 J/mol·K), *T* is the temperature (*K*), *C*_s_ (mg/L) is the amount adsorbed on the solid at equilibrium, and *C*_e_ is the concentration (mg/L) at equilibrium. Δ*G* ^0^ is given by eq 11, and the values of Δ*H* ^0^ and Δ*S* ^0^ are calculated from the slope and intercept of the plot of ln­(*K*_c_) versus 1/*T*.

Moreover, using a scanning electron microscope (SEM; Jeol JSM-6390 LV, Tokyo, Japan), the surface morphology of the products was examined. Fourier Transform Infrared Spectroscopy (FTIR) (PerkinElmer, New York, NY) was used to investigate the surface chemical bonds and functional groups. Characterization measurements were applied to many samples, and the results were consistently obtained and correlated with all other nondisplayed data. Consequently, the findings that are representative are presented below.

## Results and Discussion

The first factor analyzed in adsorption is the effect of the initial solution pH, which is one of the most significant factors that affect the adsorption performance. Thus, at 293 K and in the pH range of 2.0 to 11.0, with an initial concentrations of 5 mg/L HA, 50 mg/L DCF, and 100 mg/L RR120, and adsorbent doses of 0.5 g/L for HA and 1.0 g/L for DCF and RR120 (based on the literature^46,60,61^ and preliminary experiments), the impact of pH on the adsorption capacity of chitosan/activated carbon (Figure 1) and chitosan/activated carbon/curcumin adsorbents (Figure 2) was investigated.

**Figure 1:**
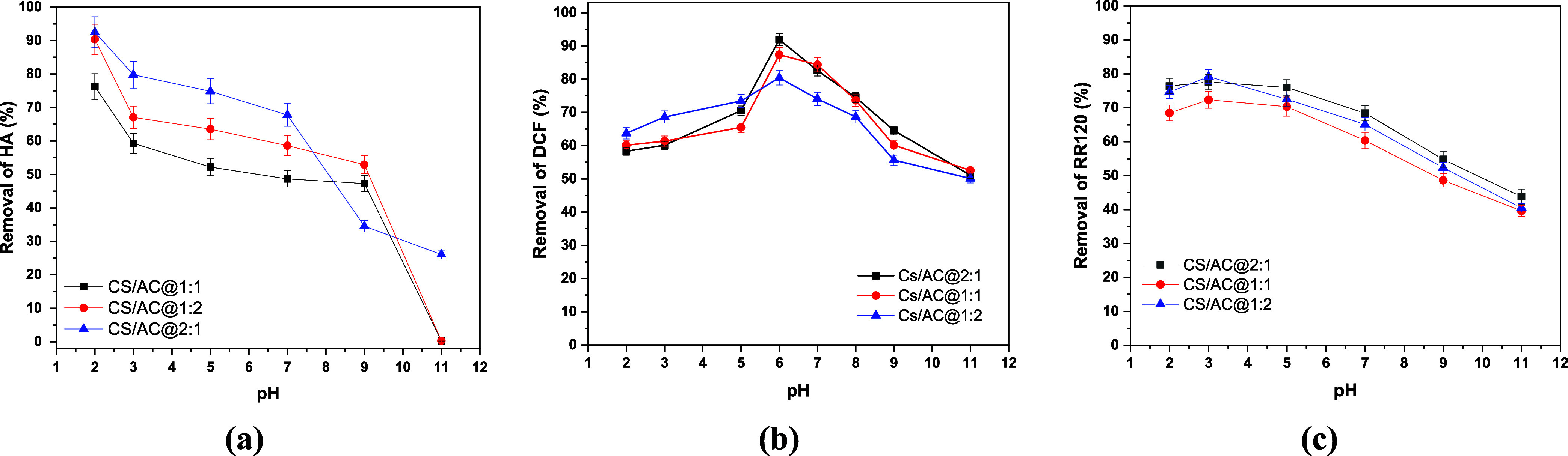
Effect of initial solution pH on the adsorption of (a) HA (*C*_0_ = 5 mg/L, dose = 0.5 g/L), (b) DCF (*C*_0_ 50 = mg/L, dose = 1.0 g/L), and (c) RR120 (*C*_0_ =100 mg/L, dose =1.0 g/L) onto chitosan/activated carbon nanoadsorbents at 293 K and 24 h contact time.

**Figure 2:**
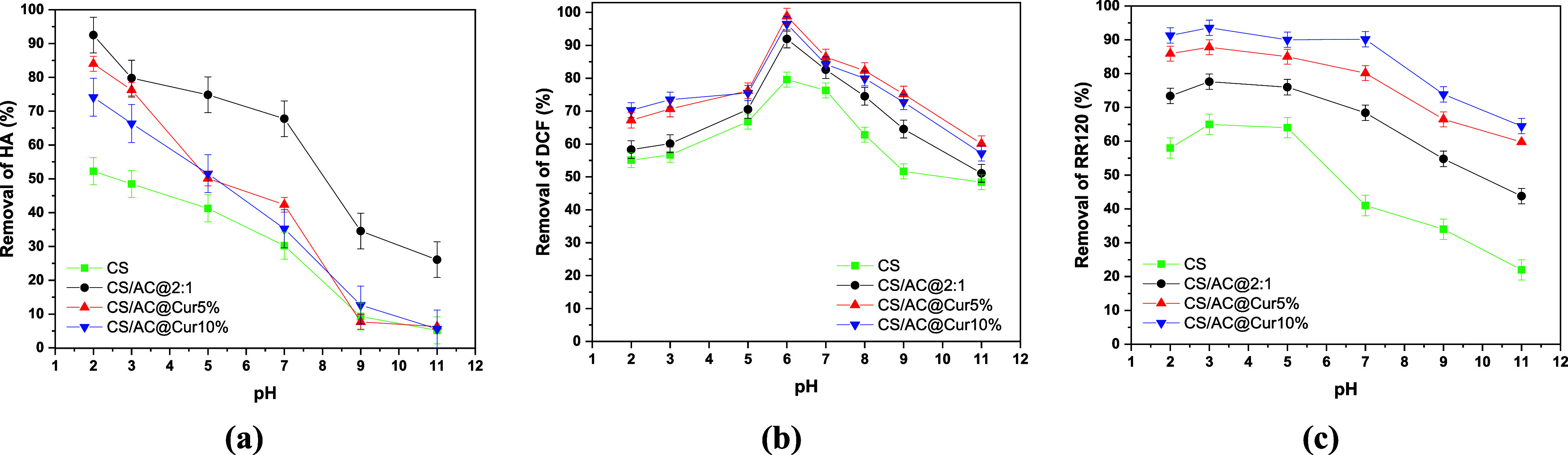
Effect of initial solution pH on the adsorption of (a) HA (*C*_0_ = 5 mg/L, dose = 0.5 g/L), (b) DCF (*C*_0_ = 50 mg/L, dose = 1.0 g/L), and (c) RR120 (*C*_0_ = 100 mg/L, dose = 1.0 g/L) onto chitosan/activated carbon/curcumin adsorbents at 293 K and 24 h contact time.

As shown in Figure 1a, the removal of HA is favored at low pH values for all of the adsorbents. In particular, the highest removal percentage of HA was achieved at pH 2.0. Initially, upon comparison of the chitosan/activated carbon derivatives (Figure 1a), a ratio of 2:1 was found to be more effective for the removal of HA, providing a 93% percentage at pH 2.0. As the pH value increases, the removal rate decreases impressively. Subsequently, the optimized CS/AC@2:1 material was compared to the most complex products containing curcumin in their structure (Figure 2a), in order to investigate whether the addition of curcumin enhanced the removal of HAs. As it appears, under the same experimental conditions, almost 84% of HA was removed by CS/AC@Cur5% and 74% by CS/AC@Cur10%. Thus, these rates are lower than 93% of CS/AC@2:1, indicating that the addition of curcumin not only did not contribute to enhancing HA removal but also reduced its rate, probably because curcumin binds to some reactive groups on the surface of CS/AC@2:1. Therefore, the optimal pH was found to be 2.0, at which the highest adsorption rates of the chitosan/activated carbon or curcumin/activated carbon nanoadsorbents were observed. Moreover, the HA solution is a mixture of compounds containing weakly acidic functional groups,^62^ which, at lower pH values, most of these groups are more adsorbable as they are in an uncharged state. Thus, the less hydrophilic part of HA will bind hydrophobic compounds more effectively, and the binding constant will increase as the pH decreases.^63^

Regarding DCF, the CS/AC@2:1 derivative exhibited the highest removal efficiency, as shown in Figure 1b. The optimal pH value was found to be 6, at which 93% of DCF was removed. The pH value seems to affect the adsorption process significantly since the Removal (%) decreases at both higher and lower pH values. This has also been observed in other studies where CS-based adsorbents were employed for the removal of DCF.^64^ This can be explained by the fact that at neutral pH, the adsorbent surface can absorb DCF drug molecules more efficiently due to electrostatic interactions.^65^ When Cur was added, the experimental results followed the same trend, with the highest adsorption efficiency at pH 6.0. In this case, Cs/AC@Cur5% exhibited the highest *R* (%) of 97%, followed by Cs/AC@Cur10% with 95%. Thus, the addition of Cur contributed to the increase of adsorption performance compared to HA removal.

In the case of RR120, the optimal pH range was found to be 2.0–5.0 for all materials, as shown in Figures 1c and 2c. This is in agreement with similar studies where CS-based adsorbents were employed for the removal of RR120.^66,67^ By increasing the Cur quantity in the adsorbents, it is clear that the removal efficiency increases, and CS/AC@Cur10% showed the highest efficiency at 93%. Thus, Cur introduces new interactions that play a vital role in the removal of this dye.

The point of zero charge (pH_pzc_) of curcumin/activated carbon adsorbents was calculated within a pH range of 2–10 ± 0.1 by plotting a relative curve in contradiction of ΔpH vs pH_initial_ using the pH drift method.^68^ The pH_pzc_ is the point at which the surface charge of the material becomes neutral.

The repulsion between HA and the negative charge on the adsorbent surface caused the efficiency of HA adsorption to decrease as the pH increased. Due to the protonation of acidic groups on the surface, the adsorbent surface becomes positively charged when the adsorption pH is less than the point of zero charge (pH_pzc_) of the adsorbent, which was found to be 6.58 for CS/AC@2:1, 6.18 for CS/AC@Cur5%, and 6.51 for CS/AC@Cur10%. On the contrary, deprotonation makes HA negatively charged, which causes electrostatic interactions to occur between the adsorbed medium and the adsorbent. Surface complexation is most likely the main mechanism controlling the removal of HA by a particular adsorbent. However, at pH levels above pH_pzc_, the adsorption efficiency decreased due to the repulsion between the negatively charge surfaces.

In the case of DCF, the optimal pH was found to be 6.0 for all of the adsorbents. At this pH value, the adsorbents are positively charged, while DCF is in the form of anions (p*K*_a_ = 4).^69^ Therefore, the main adsorption mechanism involves electrostatic attraction between the adsorbent and adsorbate. Other lesser interactions are also present, which include H-bonding (Yoshida), π–π stacking, and hydrophobic interactions, due to the presence of Cur and AC.^70^

On the other hand, the dye RR120 is anionic in the whole pH range since sodium dissociates in aqueous solutions as follows

$$R-SO_{3}\mathrm{Na}\to R-SO_{3}^{-}+Na^{+}$$

Thus, electrostatic interactions were the main adsorption mechanism, and the optimum pH value was found to be 3.0. In the Cs/AC@Cur derivatives, the Removal (%) remains relatively high, even at higher pH values. This indicates that other weaker interactions strongly contribute to the process, which include π–π stacking, hydrophobic interactions, as well as H-bonding (Yosida) and n−π interactions.^44,71^

Another important factor is the effect of the adsorbent dosage, which was analyzed. Batch experiments were carried out to determine the potential of a certain material for removing organic pollutants. In the experiments carried out, the adsorption of HA from aqueous solutions was examined at pH 2.0, while for DCF and RR120 at pH 6.0 and 3.0, respectively. The temperature was 293 K, and the contact time was 24 h, with the dosage of the materials CS/AC@Cur5% and CS/AC@Cur10% varying according to the effectiveness. In deionized water, adsorbent doses of 0.2, 0.5, 0.8, and 1.0 g/L were used. Figure 3a shows that with the increase of the adsorbent dosage, the percentage removal of HA increased, achieving a removal of more than 80% and around 80% for CS/AC@Cur5% and CS/AC@Cur10%, respectively (using 0.8 g/L). Doses of 0.2 or 0.5 g/L achieved similar results. However, a dosage of 0.5 g/L contributed to a similar removal as the dosage of 0.8 g/L. Consequently, a dosage of 0.5 g/L was chosen for further research, as it can be effective for removing HA. On the contrary, Figure 3b,c shows that the adsorption efficiency increases with increasing adsorbent dosage. Thus, for DCF and RR120, a dosage of 1.0 g/L was selected for the remaining experiments.

**Figure 3:**
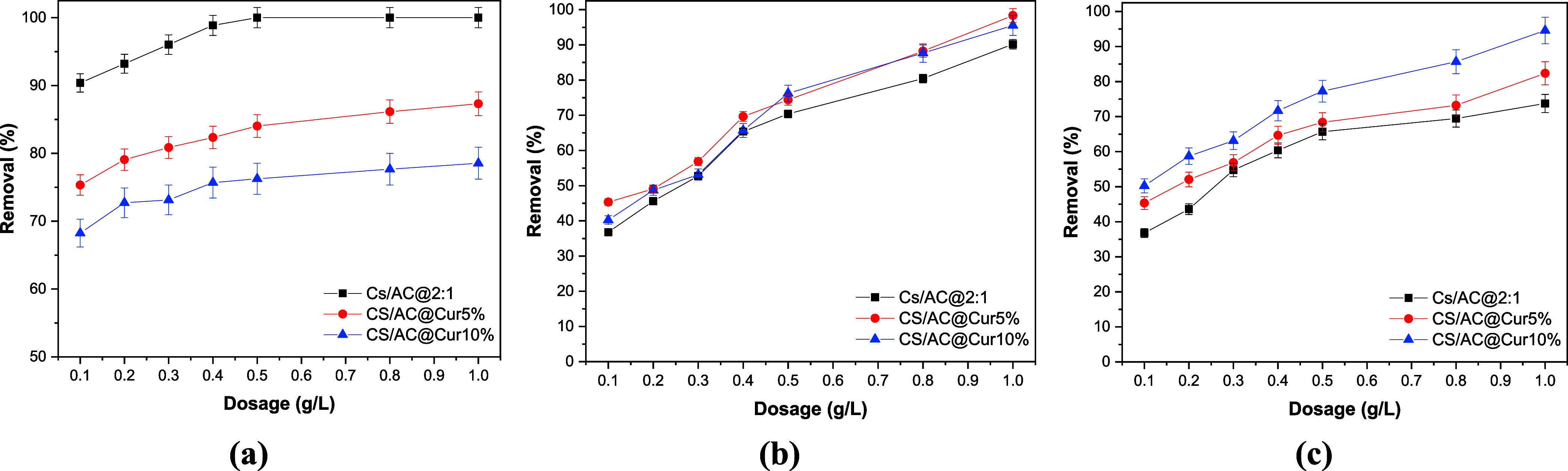
Effect of dosage on the adsorption of (a) HA (*C*_0_ = 5 mg/L, pH = 2.0 ± 0.1), (b) DCF (*C*_0_ = 50 mg/L, pH = 6.0 ± 0.1), and (c) RR120 (*C*_0_ = 100 mg/L, pH = 3.0 ± 0.1) onto chitosan/activated carbon/curcumin nanoadsorbents at 293 K for 24 h contact time.

Adsorption kinetics play an important role in understanding this phenomenon. Therefore, in Figure 4a–c, the effect of contact time on the adsorption of organic compounds onto chitosan/activated carbon derivatives in the range of 5–1440 min (24 h) is presented. All other factors, such as the initial concentration, the adsorbent dosage, pH, and temperature *T* = 293 K, were held constant and at optimal conditions during the research. In the case of HA, as the contact time increases, the effectiveness of the removal from the aqueous solution increases and eventually reaches a plateau (after 180 min). The results showed that during the first 60 min, there was rapid HA adsorption of up to 70% removal, suggesting that the surface was easily accessible for adsorption. The removal rate increased over time between 60 and 180 min, reaching 75 and 80% for CS/AC@Cur10% and CS/AC@Cur5%, respectively. Equilibrium was reached after 1440 min. A contact time of 2 h (120 min) was selected as the optimal time for further research. For DCF, a plateau was reached in 90 min of the process, with a rapid increase in the period of 0–60 min. This implies that during the early stages of contact time, when many surface sites are accessible for adsorption, DCF adsorption occurs rapidly. Finally, for RR120, a fast adsorption occurred in the first hour, and the performance gradually stabilized. In this case, equilibrium was achieved at 2 h, which was selected as the optimal time.

**Figure 4:**
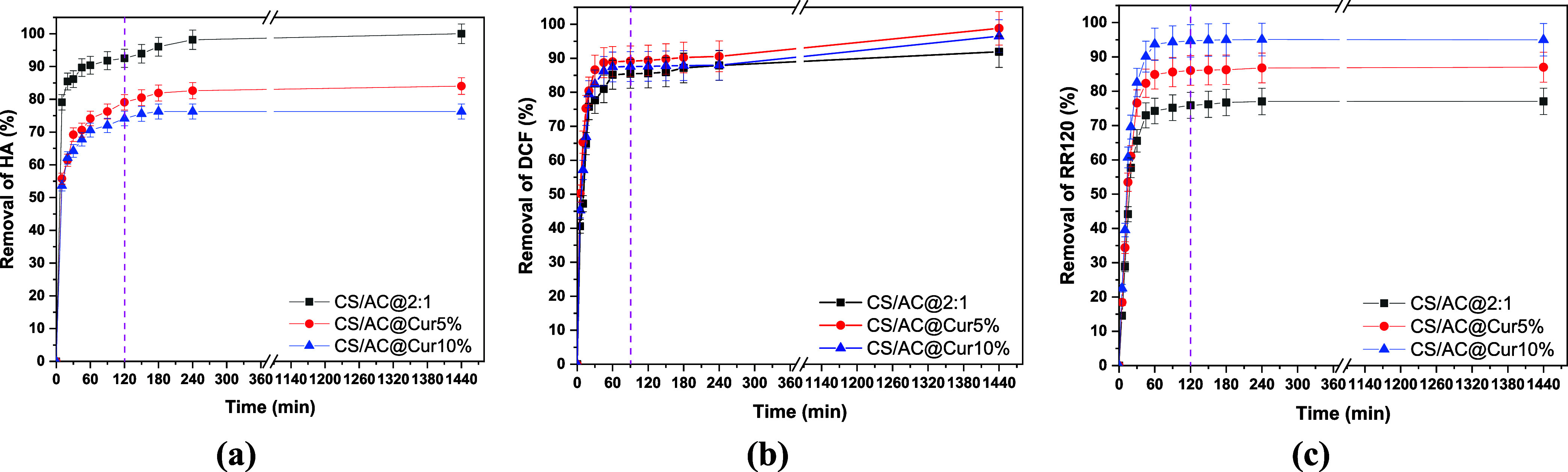
Effect of contact time on the adsorption of (a) HA (pH = 2.0 ± 0.1, *C*_0_ = 5 mg/L, dose = 0.5 g/L), (b) DCF (pH = 6.0 ± 0.1, *C*_o_ = 50 mg/L, dose =1.0 g/L) and (c) RR120 (pH = 3.0 ± 0.1, *C*_o_ =100 mg/L, dose =1.0 g/L) onto chitosan/activated carbon/curcumin adsorbents at 293 K.

The applicability of the nonlinear PFO and PSO kinetic models was investigated (Figure 5), and the obtained kinetic parameters are shown in Table 2. The rate of adsorption and the mechanism controlling the entire process can be understood through the application of adsorption kinetics. Using pseudo-first-order (PFO) and pseudo-second-order (PSO) kinetic models, the kinetics of the adsorption of organic contaminants on chitosan/activated carbon/curcumin adsorbents were interpreted.

**Figure 5:**
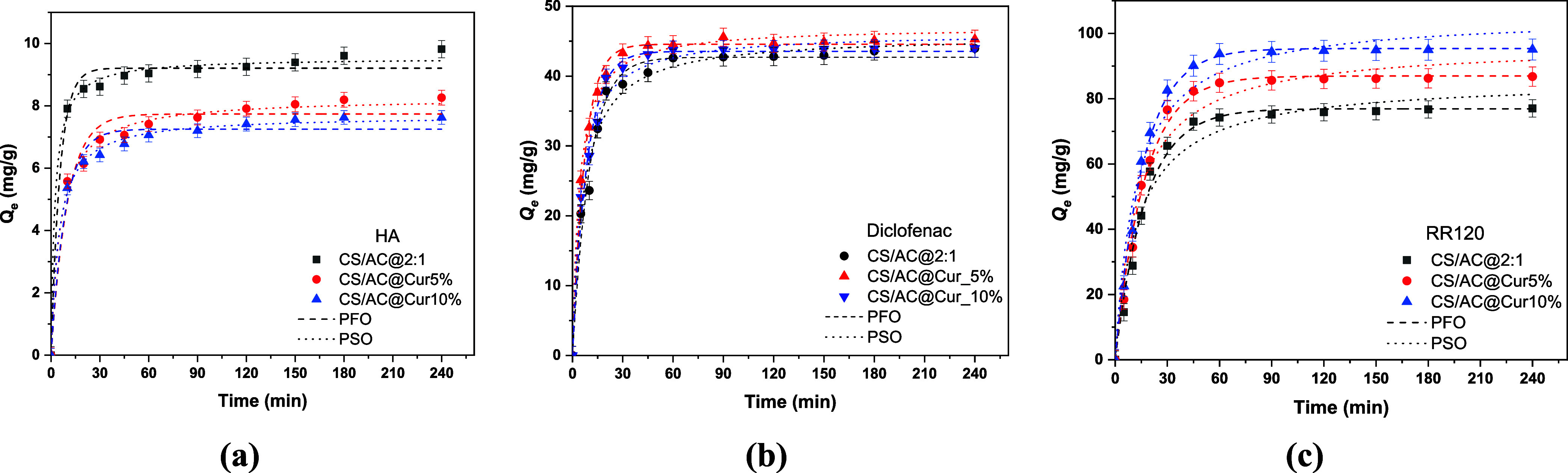
Kinetic pseudo-first and pseudo-second order models for the adsorption of (a) HA (pH = 2.0 ± 0.1, *C*_0_ = 5 mg/L, dose 0.5 = g/L), (b) DCF (pH = 6.0 ± 0.1, *C*_o_ = 50 mg/L, dose = 1.0 g/L), and (c) RR120 (pH = 3.0 ± 0.1, *C*_o_ = 100 mg/L, dose = 1.0 g/L) onto chitosan/activated carbon/curcumin nanoadsorbents at 293 K.

**Table 2:** Pseudo-First- and Pseudo-Second-Order Kinetic Parameters for the Adsorption of Organic Compounds onto Chitosan/Activated Carbon and Chitosan/Activated Carbon/Curcumin Nanoadsorbents

		pseudo-first-order kinetic parameters			pseudo-second-order parameters		
adsorbent	*Q*_e,exp_ (mg/g)	*K*_1_ (1/min)	*Q*_e.cal_ (mg/g)	*R* ^2^	*K*_2_ (L/mg·min)	*Q*_e,cal_ (mg/g)	*R* ^2^
HA							
CS/AC@2:1	10.00	0.2218	9.13	0.788	0.0452	9.54	0.960
CS/AC@Cur5%	8.40	0.1005	7.74	0.960	0.0210	8.26	0.993
CS/AC@Cur10%	7.63	0.1150	7.25	0.972	0.0272	7.69	0.997
DCF							
CS/AC@2:1	43.95	0.0972	42.69	0.982	0.0034	45.82	0.982
CS/AC@Cur5%	45.28	0.1392	44.57	0.992	0.0053	47.02	0.994
CS/AC@Cur10%	43.99	0.1152	43.55	0.987	0.0043	46.23	0.988
RR120							
CS/AC@2:1	77.04	0.0578	76.91	0.989	0.0009	85.85	0.959
CS/AC@Cur5%	86.78	0.0597	86.95	0.993	0.0008	96.92	0.964
CS/AC@Cur10%	95.08	0.0623	95.38	0.996	0.0008	105.54	0.969

By comparing the *R* ^2^ values (>0.95) shown in Table 2, it was determined that the HA adsorption investigated on the surface of both tested adsorbents was better suited to the pseudo-second-order model. The PSO model and its nonlinear forms are also shown in Figure 5a–c. Furthermore, the kinetic parameters demonstrated that nearly all of the *Q*_e,cal_ values derived from the equations were equivalent to the actual values obtained during HA adsorption studies on chitosan/activated carbon/curcumin adsorbents. As can be observed, there is little difference in the coefficient of determination (*R* ^2^) between the pseudo-second and pseudo-first orders. However, both exceed 95%, indicating that they accurately capture the kinetic data. However, the adsorption kinetic analysis indicated that the adsorption was closer to chemisorption. Regarding DCF, both kinetic models had similar *R* ^2^ values, but PSO had a slightly higher value. This indicates that both physisorption and chemisorption strongly affect the adsorption process. This is in agreement with the pH analysis results. However, chemisorption seems to be the dominant model. In the case of RR120, it seems that PFO has the best fit, exhibiting an *R* ^2^ > 0.98 for all adsorbents tested. The kinetic parameters also showed that the *Q*_e,cal_ values are close to the experimental data obtained. Thus, physisorption plays a significant role in the removal of RR120. This can be explained by the fact that Cur is decorated on the adsorbent surface and primarily interacts through π–π stacking and hydrophobic interactions.

To avoid dispersion of the fitting parameters, the values of *k*_h_ and *y* that provide the overall best fit for each examined solute are chosen. These values are *y* = 2 and *k*_h_ = 2 (g/mg)^2^ for HA, and *k*_h_ = 0 for DCF and RR120. A comparison between the experimental data and model results regarding the evolution of the actually measured quantity (solute concentration) is presented in Figure 6. There is a small discrepancy (<2 mg/L) in values close to equilibrium (large time). This is due to the small deviation of the Langmuir isotherm from the experimental equilibrium data. The values of *K*_o_ resulting from the fitting procedure are 1, 0.6, 0.6 min^–1^ (HA); 0.02, 0.023, 0.02 min^–1^ (DCF); and 0.03, 0.02, 0.014 min^–1^ (RR120) for the three optimum materials used, i.e., CS/AC@2:1, CS/AC@Cur5%, and CS/AC@Cur10%, respectively. The difference in the *K*_o_ values between HA and the other two solutes is due to the presence of an inhibition factor in the case of HA. The addition of curcumin appears to reduce the adsorption kinetic rates for HA and RR120, but it had no effect on DCF.

**Figure 6:**
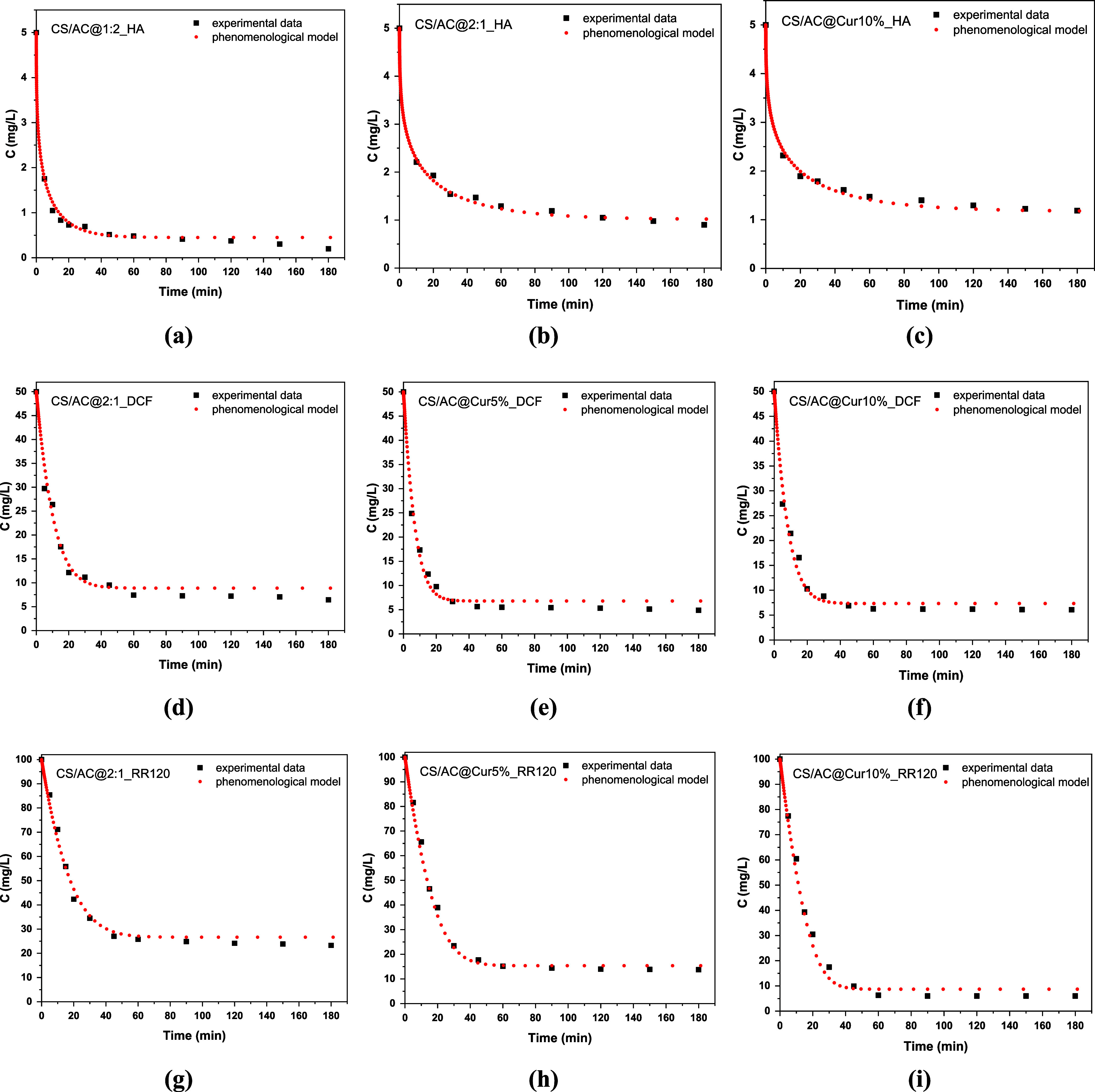
Comparison between experimental (symbols) and model (dashed line) results for the evolution of the bulk solute concentration for chitosan/activated carbon/curcumin adsorbents, for (a–c) HA, (d–f) DCF, and (g–i) RR120 removal.

All of the above experimental findings were further examined by fitting the equilibrium results into Langmuir and Freundlich isotherm models at optimum conditions. The collected experimental data were fitted to these models, and the resulting plots are displayed in Figure 7a–i, while Table 3 summarizes the respective equilibrium constants and coefficients.

**Figure 7:**
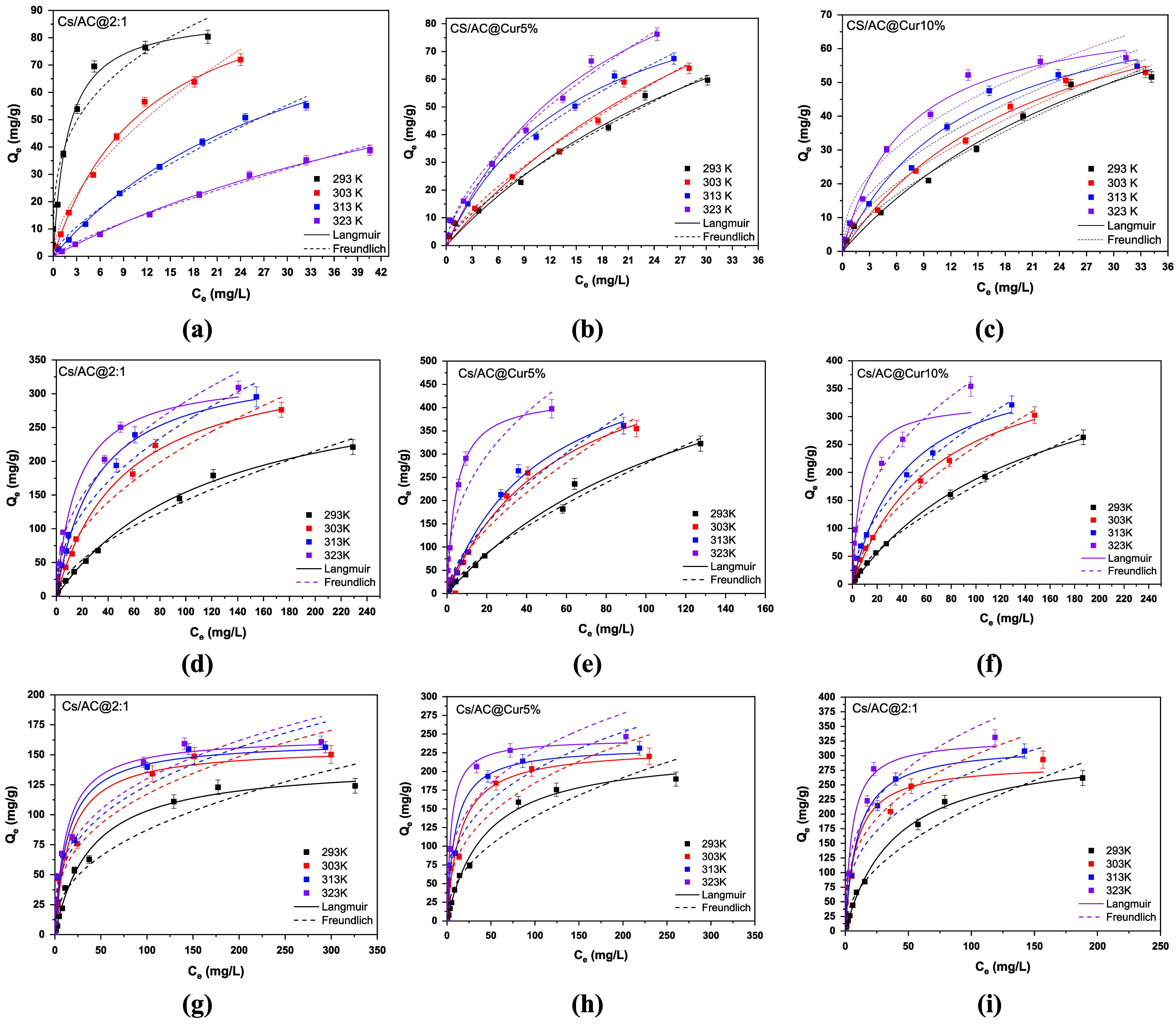
Langmuir and Freundlich isotherm models for the adsorption of (a–c) HA (pH =2.0 ± 0.1, *C*_o_ = 2–60 mg/L, dose = 0.5 g/L, and 2 h contact time), (d–f) DCF (pH = 6.0 ± 0.1, *C*_o_ = 5–450 mg/L, dose = 1.0 g/L, and 1.5 h contact time), and (g–i) RR120 (pH 3.0 ± 0.1, *C*_o_ = 5–450 mg/L, dose = 1.0 g/L, and 2 h contact time), onto curcumin/activated carbon nanoadsorbents at 293, 303, 313, and 323 K.

**Table 3:** Constants of the Freundlich and Langmuir Isotherm Models for the Adsorption of HA, DCF, and RR120 onto Chitosan/Activated Carbon and Chitosan/Activated Carbon/Curcumin Adsorbents

		freundlich isotherm model			langmuir isotherm model		
adsorbent	*T* (K)	1/*n*	*K*_F_ (mg/g)·(L/mg)^1/*n*^	*R* ^2^	*Q*_m_ (mg/g)	*K*_*L*_ (L/mg)	*R* ^2^
HA							
CS/AC@2:1	293	0.201	35.67	0.9416	108.99	0.144	0.9811
	303	0.578	12.08	0.9819	108.44	0.083	0.9965
	313	0.710	4.94	0.9900	116.40	0.029	0.9976
	323	0.790	2.18	0.9934	110.01	0.014	0.9974
CS/AC@Cur5%	293	0.722	5.25	0.9915	148.0	0.023	0.9883
	303	0.731	5.75	0.9809	172.3	0.022	0.9760
	313	0.575	10.57	0.9940	106.6	0.064	0.9871
	323	0.607	11.21	0.9927	136.1	0.052	0.9837
CS/AC@Cur10%	293	0.684	4.93	0.9830	111.9	0.027	0.9843
	303	0.604	6.81	0.9840	91.3	0.044	0.9885
	313	0.525	9.56	0.9728	80.8	0.072	0.9839
	323	0.454	13.88	0.9570	72.8	0.143	0.9915
DCF							
CS/AC@2:1	293	0.413	43.00	0.9732	340.56	0.008	0.9940
	303	0.461	30.94	0.9743	328.89	0.062	0.9837
	313	0.509	21.28	0.9768	349.66	0.032	0.9906
	323	0.602	8.81	0.9855	360.41	0.019	0.9897
CS/AC@Cur5%	293	0.725	9.93	0.9863	675.01	0.007	0.9913
	303	0.643	20.23	0.9529	579.94	0.018	0.9805
	313	0.633	22.66	0.9616	568.38	0.210	0.9934
	323	0.391	92.12	0.9155	428.15	0.223	0.9920
CS/AC@Cur10%	293	0.401	58.08	0.9858	327.77	0.154	0.9540
	303	0.530	25.02	0.9961	409.15	0.023	0.9877
	313	0.681	7.70	0.9958	435.29	0.014	0.9963
	323	0.595	15.95	0.9953	465.11	0.007	0.9990
RR120							
CS/AC@2:1	293	0.415	12.99	0.9416	143.07	0.026	0.9917
	303	0.341	24.33	0.9384	156.66	0.063	0.9686
	313	0.330	27.15	0.9366	161.72	0.072	0.9622
	323	0.315	30.32	0.9333	165.07	0.083	0.9473
CS/AC@Cur5%	293	0.456	17.16	0.9369	229.00	0.234	0.9929
	303	0.360	35.10	0.9370	231.92	0.067	0.9754
	313	0.324	45.39	0.9201	233.81	0.109	0.9350
	323	0.296	57.86	0.8943	243.58	0.214	0.9333
CS/AC@Cur10%	293	0.305	84.78	0.9463	329.33	0.197	0.9652
	303	0.316	69.77	0.9647	317.61	0.026	0.9973
	313	0.331	59.10	0.9743	286.40	0.119	0.9806
	323	0,487	22.34	0.9607	318.40	0.105	0.9695

The Freundlich isotherm model (*R* ^2^ = 0.99) more accurately reflected the adsorption of HA on both CS/AC@Cur10% and CS/AC@Cur5%, based on the correlation values (*R* ^2^). The Freundlich isotherm model states that adsorption is a chemical process when *n* is <1, a linear process when n is equal to 1, and a physical process when *n* is greater than 1. In this study, as depicted in Figure 7 and Table 3, which display several isotherm parameters, the value of n was found to be >1, indicating that the adsorption of HA onto both adsorbents is a favorable physical process. A higher value of *n* suggests a stronger interface between the adsorbent surface and HA. Furthermore, the effectiveness of the adsorption performance increases with higher values of *K*_F_, which reach values of 11.64 and 10.29 for CS/AC@Cur5% and CS/AC@Cur10% at 323 K, respectively. It should be noted that the Freundlich adsorption isotherm model applies to heterogeneous surfaces.

In contrast, both DCF and RR120 fit a better Langmuir isothermal model. The Langmuir isotherm model implies that adsorption occurs on homogeneous surfaces. This isotherm model also assumes that the adsorption–desorption process is reversible.^72^ Cs/AC@Cur5% exhibits higher *K*_L_ values than the other adsorbents for both of these pollutants. This indicates that it has a stronger binding activity, even at lower concentrations. Lastly, it can be observed that Cur enhanced significantly the adsorption capacity of the materials. For DCF, CS/AC@2:1 exhibits a *Q*_m_ of 328.89 mg/g at 303 K, which then increases to 579.94 and 409.15 mg/g for CS/AC@Cur5% and CS/AC@Cur10%, respectively. In the case of RR120, CS/AC@2:1 exhibits a *Q*_m_ of 156.66 mg/g at 303 K, which then increases to 231.92 mg/g for CS/AC@Cur5% and 317.61 mg/g for CS/AC@Cur10%.

The thermodynamic properties and nature of the system were analyzed by plotting ln­(*K*_c_) vs 1/*T* to determine the values of Δ*H* ^0^ and Δ*S* ^0^. When the Δ*G* ^0^ number is positive, it means that external energy is needed for the process to occur, and when it is negative, it means that the process is spontaneous.^73^ The negative Δ*G* ^0^ values, as shown in Table 4, indicate that the adsorption of all of the studied organic pollutants occurs spontaneously for all of the adsorbents in the present study. It is clear that the removal rate tends to increase with increasing temperatures. For CS/AC/Cur@5% and CS/AC/Cur@10%, the Δ*H* ^0^ values for HA were found to be 24.880 and 16.823 kJ/mol, respectively, suggesting that the adsorption process was endothermic. Additionally, the Δ*H* ^0^ values for DCF were found to be 26.953 and 40.954 kJ/mol for CS/AC/Cur@5% and CS/AC/Cur@10%, respectively. In the case of RR120, the corresponding values were 45.192 kJ/mol and 48.364. These lower values for CS/AC@Cur5% indicate that the adsorption on this material is stronger compared to CS/AC@Cur10%

**Table 4:** Thermodynamic Parameters for the Adsorption of HA (pH = 2.0 ± 0.1, *C*_o_ = 5 mg/L, dose = 0.5 g/L, and 2 h Contact Time), DCF (pH = 6.0 ± 0.1, *C*_o_ = 100 mg/L, dose = 1.0 g/L, and 1.5 h Contact Time), and RR120 (pH = 3.0 ± 0.1, *C*_o_ = 100 mg/L, Dose = 1.0 g/L and 2 h Contact Time) onto Chitosan/Activated Carbon and Chitosan/Activated Carbon/Curcumin Nanoadsorbents at 293, 303, 313, and 323 K

adsorbent	*T* (K)	Δ*G* ^*0*^ (kJ/mol)	Δ*H* ^*0*^ (kJ/mol)	Δ*S* ^*0*^ (kJ/mol·K)	*R* ^2^
HA					
CS/AC@2:1	293	–4.945	51.614	0.1593	0.9368
	303	–3.352			
	313	–1.760			
	323	–0.167			
CS/AC/Cur@5%	293	–3.583	24.880	0.0971	0.8787
	303	–4.554			
	313	–5.525			
	323	–6.497			
CS/AC/Cur@10%	293	–3.854	16.823	0.0706	0.9883
	303	–4.560			
	313	–5.265			
	323	–5.971			
DCF					
CS/AC@2:1	293	–1.81	58.371	0.206	0.9819
	303	–4.31			
	313	–5.91			
	323	–7.86			
CS/AC/Cur@5%	293	–3.54	26.953	0.105	0.8165
	303	–5.30			
	313	–5.60			
	323	–11.34			
CS/AC/Cur@10%	293	–2.37	40.954	0.128	0.9904
	303	–4.08			
	313	–5.32			
	323	–10.14			
RR120					
CS/AC@2:1	293	–1.27	30.821	0.110	0.9171
	303	–2.89			
	313	–3.45			
	323	–3.97			
CS/AC/Cur@5%	293	–2.57	45.192	0.163	0.9783
	303	–4.58			
	313	–5.82			
	323	–8.84			
CS/AC/Cur@10%	293	–4.14	48.364	0.181	0.8115
	303	–7.25			
	313	–7.69			
	323	–9.68			

Moreover, the reusability of chitosan/activated carbon/curcumin adsorbents for the removal of HA, DCF, and RR120 was investigated through adsorption–desorption experiments. Initially, desorption experiments were performed using different aqueous eluents with pH values ranging from 3–12. According to Figure 8a, the desorption of the 3 studied organic pollutants was favored by alkaline conditions. In particular, the highest values were achieved at pH 12 at 94.7, 89.5, and 86.2% for HA, DCF, and RR120, respectively. However, because at pH 10 relatively similar desorption efficiencies were achieved (89.5, 79.3, 78.3 for HA, DCF, and RR120, respectively), it was selected as the optimal eluant since these conditions are not extreme. The materials could be reused effectively for up to 6 cycles after successful regeneration by 0.01 M NaOH. As shown in Figure 8b,c, a gradual decrease in regeneration ability was observed within each cycle, indicating that the chitosan/activated carbon/curcumin adsorbents proposed in this study can be successfully reused multiple times for the removal of the studied pollutants. Particularly, for HA removal by the optimum CS/AC@2:1 adsorbent, in the first cycle, there was 92.5% removal, up to the fourth cycle, where the removal was still high (80.0%), falling to 62.1% at the end of the sixth cycle. In the case of DCF removal by CS/AC/Cur@5%, the relative percentages were 89.4% in the first cycle, 81.3% in the fourth cycle, presenting only a 9% reduction in efficiency, up to the sixth cycle (75.3%). For RR120, the removal was initially about 94.5% (1st cycle) and 84.2% in the fourth cycle, while it reached 75.2% after the sixth cycle by the CS/AC/Cur@10% application. Therefore, an overall reduction of approximately 15–20% was observed, possibly due to the mass loss after each cycle, confirming that these adsorbents can be used effectively for up to six cycles without their performance being significantly declined. According to Figure 8e, no leaching of the material compounds was detected in the water. The spectra of the 3 adsorbents matched the spectrum of DI water, which was used for the batch experiments. In addition, no weight loss of the composite was detected (<0.5% for each case). Thus, this study highlights the rigidity of the materials resulting from the strong cross-linking.

**Figure 8:**
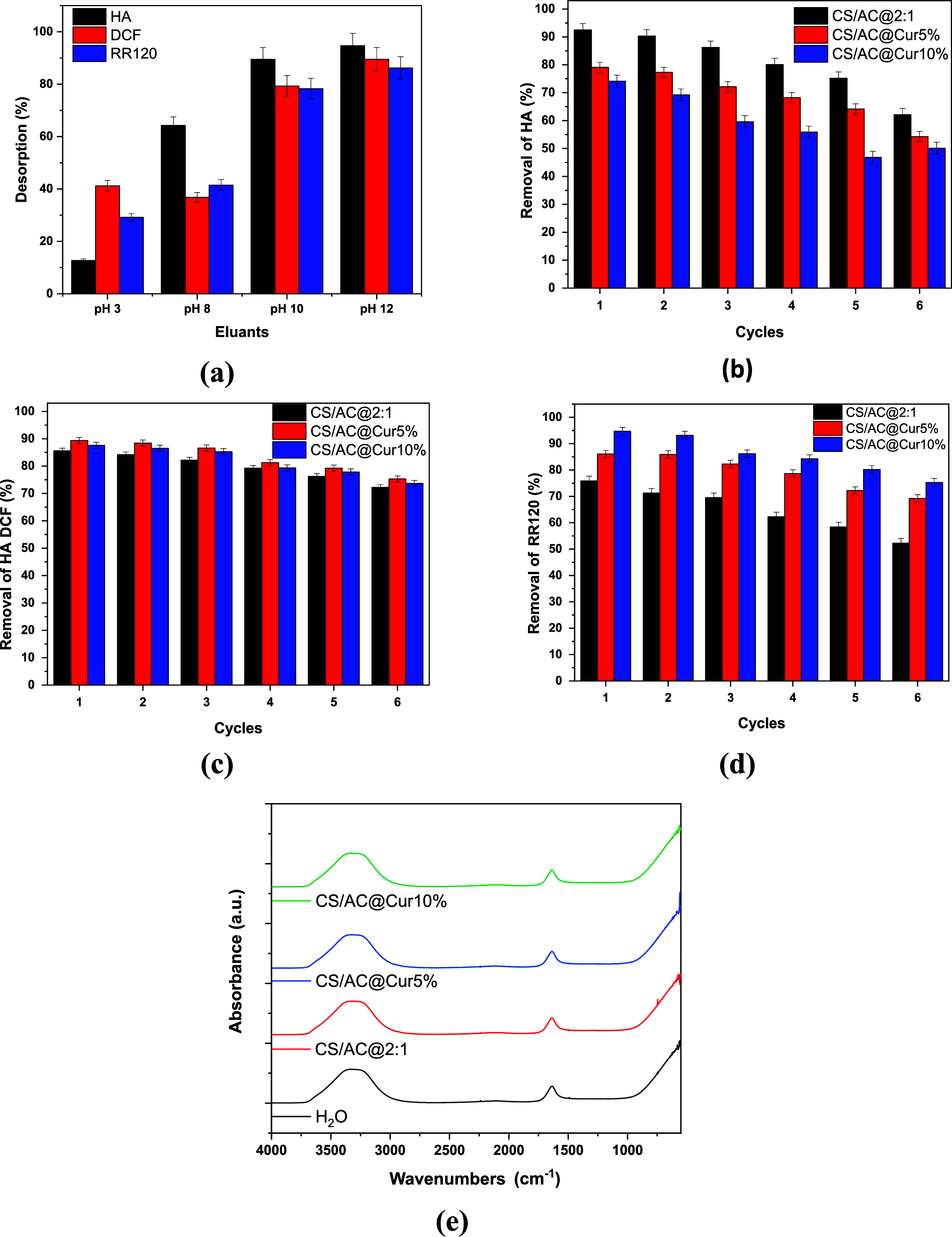
(a) Desorption of organic pollutants by aqueous eluents. Adsorption of (b) HA, (c) DCF, and (d) RR120 onto chitosan/activated carbon/curcumin nanoadsorbents for six adsorption–desorption cycles after regeneration by using 0.01 M NaOH at 293 K and 2 h contact time, and (e) leaching examination after the 1st cycle.

As noted previously, it is important to check the physicochemical properties of the prepared materials. Therefore, Scanning Electron Microscopy (SEM) (Jeol JSM-6390 LV, Japan scanning electron microscope) and Fourier Transform Infrared Spectroscopy (FTIR, PerkinElmer, New York, NY) were used to study the surface of the optimum adsorbents pre- and postadsorption. Characterizations were performed on several samples, and the typical and repeatable results are presented.

The SEM images presented in Figure 9 show the microstructures of the adsorbents before and after the adsorption process. As can be seen, the surface of the CS/AC@2:1 adsorbent (Figure 9a) exhibited a highly heterogeneous and irregular surface with a few random wavy points and microfibrils. This type of morphology was also observed in other similar studies.^74–76^ The primary causes of shape irregularities are sample grinding and varying degrees of grafting. After adsorption of HA (Figure 9d), the adsorbent appears to have a rough surface with thicker pores as they are partially covered by HA molecules. In addition, the SEM images of the other two adsorbents, CS/AC@Cur5% and CS/AC@Cur10% (Figure 9b,c), show a cleaner and smoother surface and less porous structure than that of CS/AC@2:1. This may exhibit a lower adsorption capacity due to the reduced number of active sites available. These minor variations are observed because all materials were synthesized using a relatively comparable technique (blending, washing/extraction, and drying). A similar structure with thicker pores on the surface of both CS/AC@Cur5% and CS/AC@Cur10% (Figure 9e,f, respectively) is also observed after adsorption, leaving some areas smooth, particularly where HA molecules are adsorbed. After adsorption of DCF (Figure 9g–i), the materials retained their structural integrity, exhibiting again a smooth surface, which is similar to that prior to adsorption. In the case of RR120 (Figure 9j–l), the adsorption of this dye had the greatest impact on the surface of the materials, as there were some cracks in certain areas. However, the smooth surface of all the materials was retained. The smoothening of the surface of these 3 materials in all the pollutants studied is an indicator of total filling of the adsorbent surface, confirming the adsorption phenomena.^77^

**Figure 9:**
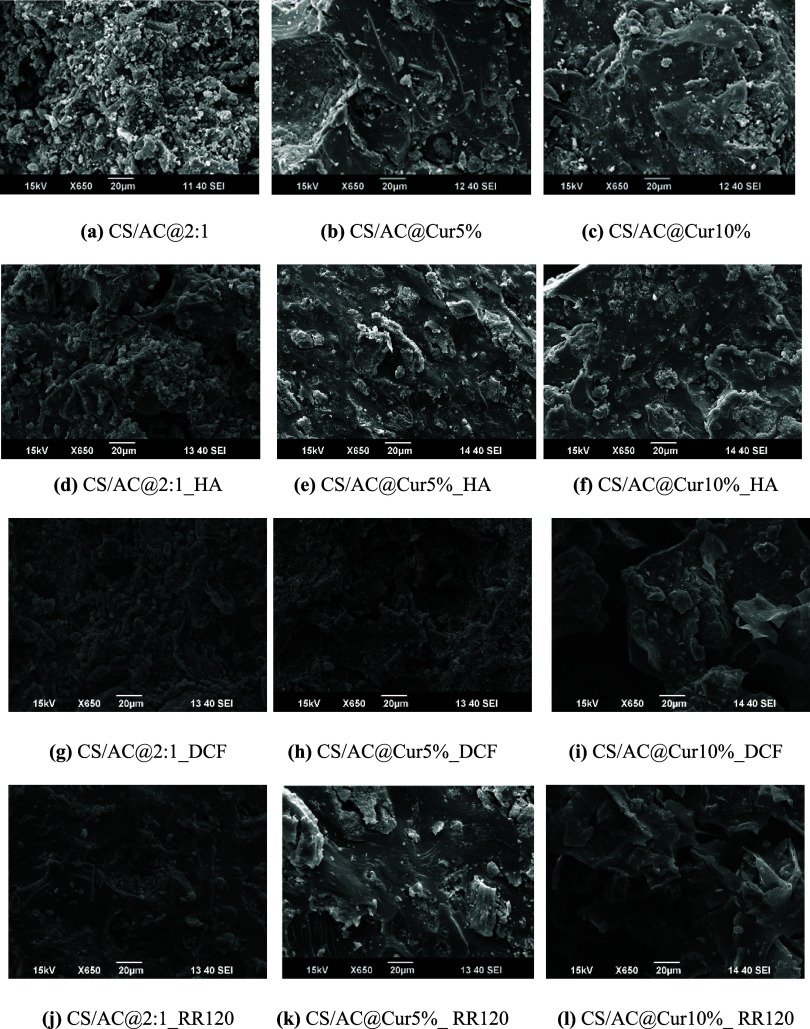
SEM images of CS/AC@2:1, CS/AC@Cur5%, and CS/AC@Cur10% adsorbents (a–c) before and after (d–f) HA, (g–i) DCF, and (j–l) adsorption.

The FTIR spectra of the three adsorbents CS/AC@2:1, CS/AC@Cur5%, and CS/AC@Cur10% before and after HA adsorption in the 4000–550 cm^–1^ region were determined and compared to obtain information on the possible interactions between the adsorbent and HA. The comparative spectra are shown in Figure 10a–c. In particular, Figure 10a–c compares the three adsorbents before and after the adsorption of each contaminant. In addition, FTIR analysis was also conducted on the composites that were not subjected to ultrasound treatment during the synthesis procedure, and the results are presented in Figure S1. As can be seen, there is no oxidative damage to the composites, which have an almost identical structure to those that have been sonicated.

**Figure 10:**
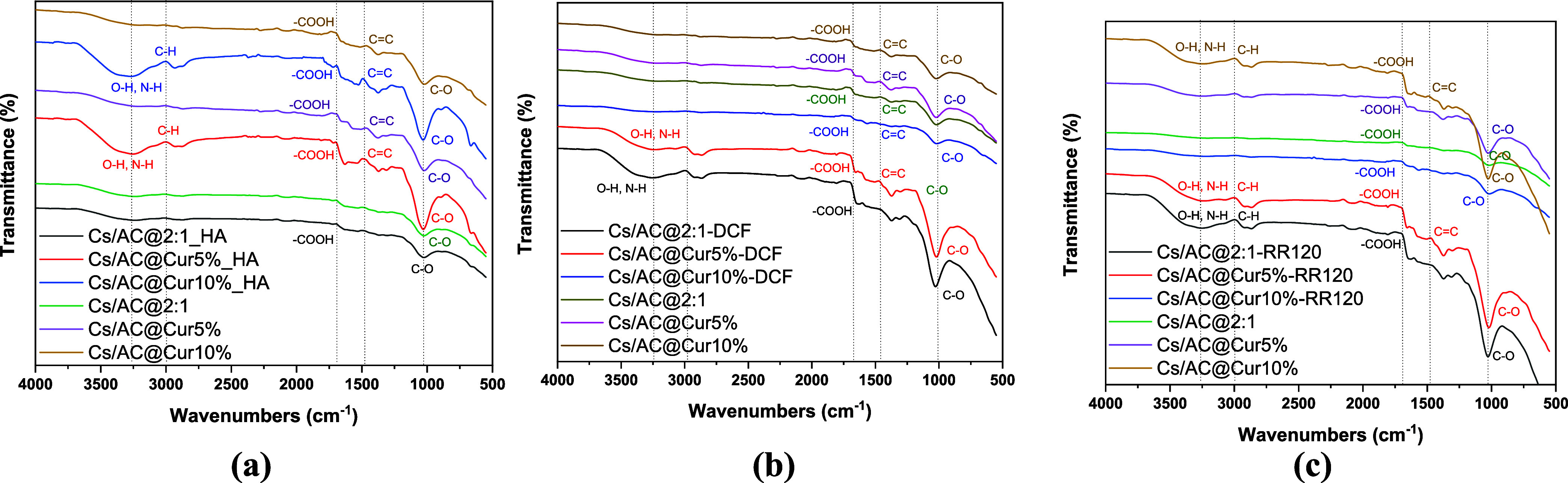
FTIR spectra of (a) HA, (b) DCF, and (c) RR120 before and after adsorption.

As shown in Figure 10a, a band is formed at around 3291 cm^–1^ as a result of the O–H and N–H stretching vibrations;^78^ these are the characteristic peaks of the amine and hydroxyl groups present in chitosan. The C–H bond vibrations are represented by the bands at 2930 and 2968 cm^–1^. The C–O–C group is responsible for the peak at 1024 cm^–1^, while the N–H bending of amide II is associated with the bands at 1649 and 1553 cm^–1^. A peak representing the stretching of the C–N bond was observed at 1372 cm^–1^. After HA adsorption, some minor changes were observed in the wavenumbers of specific bonds. The FTIR spectra of the curcumin samples (Figure 10b,c) also highlight the presence of bonds from these groups as follows: the band centered at 3425 cm^–1^ indicates the presence of O–H bonds attached to the benzene rings, the bands in the region 3050–2800 cm^–1^ indicate the presence of C–H bonds from aliphatic components and =C–H in the benzene ring.^79^ At 1800–800 cm^–1^, the main peaks are dedicated to C=O from the carbonyl groups at 1721 and 1629 cm^–1^, C=C from the phenyl group at 1514 cm^–1^, C–H from the aliphatic groups at 1470–1380 cm^–1^, and C–O from the ether groups, shown at 1200–1000 cm^–1^. This peak at 1260 cm^–1^ is related to the bending vibrations of hydroxyls existing in chitosan and was observed in all spectra (Figure 10a–c).^80^ The aforementioned bands found in the spectra of samples containing chitosan are also reported in the literature.^80,81^ As can also be observed, the stretching vibrations around the aromatic C=C bonds of the keto and enolic forms of curcumin are observed at around 1514 cm^–1^, present only in the spectra of curcumin-containing adsorbents^82^ and becoming more intense after HA adsorption due to the entrance of −COOH groups from its structure.^83,84^

The FTIR spectra of the postadsorption of DCF confirm the successful integration of DCF. No new peaks appear. There are mainly shifts in the region of the O–H/N–H stretching (3000–3500 cm^–1^), indicating electrostatic interactions or H-bonding between the adsorbents and DCF. In addition, the reduced transmittance intensity in the samples loaded with DCF also indicates strong binding of the pollutant. All these results collectively reveal physical adsorption controlled by surface interactions rather than chemical bonding. Similarly, in the FTIR spectra of post-RR120, no new peaks appear. Only a few shifts in the peaks of the materials were observed prior to adsorption. The peaks in the O–H/N–H stretching regions (3000–3500 cm^–1^) indicate H-bonding or electrostatic interactions between RR120 and −OH/–NH groups of the CS/AC and CS/AC@Cur composites. The reduced transmittance intensities in the RR120-adsorbed samples further confirm adsorption, with CS/AC@Cur10%-RR120 having a higher adsorption efficiency, likely due to greater curcumin loading providing more binding sites. Thus, the absence of new chemical bonds and the dominance of peak shifts/intensity changes indicate that adsorption is predominantly physisorption, owing to surface interactions instead of covalent bonding.

Table 5 presents the determined values of the Brunauer–Emmett–Teller (BET) surface area (*S*_BET_), Barrett–Joyner–Halenda (BJH) average pore diameter, and total pore volume at *P*/*P*_o_ = 0.985. Regarding the specific surface area, the addition of Cur resulted in higher values, with Cs/AC@Cur10% having the largest value of 42.6 m^2^/g, which is an increase of ∼8 m^2^/g compared to the Cs/AC@2:1. The pore size distributions (PSD) of the adsorbents were similar, implying no significant differences in the structures of the materials. The total pore volume seems to be affected more by the incorporation of Cur on the Cs/AC@2:1 surface, as it almost doubles its value from 0.012 to 0.019 and 0.021 cm^3^/g for Cs/AC@Cur5% and Cs/AC@Cur10%, respectively. Based on N_2_ isotherms of the composites (Figure S2), they exhibit a Type II isotherm, according to IUPAC, suggesting macroporous materials.

**Table 5:** Physical Properties of Optimal Materials

physical properties	Cs/AC@2:1	Cs/AC@Cur5%	Cs/AC@Cur10%
BET surface area, *S*_BET_ (m^2^/g)	34.12	37.28	42.6
pore size distribution, PSD (Å)	23.39	22.52	20.12
total pore volume, *V*_T_ (cm^3^/g)	0.012	0.019	0.021

For comparison with other Cs-based adsorbents, the optimal material for each dye was selected and compared with other results from the recent literature, and the results are summarized in Table 6. In the case of HA, Cs/AC@2:1 showed a maximum adsorption capacity of 148 mg/g, which is significantly higher than that of most of the reported materials. Only chitosan/cellulose acetate molecules managed to remove higher quantities of 184.72 mg/g using only 0.05 g/L at pH 4. For RR120, Cs/AC@Cur10% exhibits a better performance (143.07 mg/g) than chitosan–epichlorohydrin and chitosan/biomass materials, being surpassed only by a chitosan/zeolite composite (284.2 mg/g). Finally, in the case of DCF, Cs/AC@Cur5% significantly outperforms the other adsorbents since it can remove up to 675.01 mg/g at pH 6, almost 3 times more than the second-best material, chitosan–PEI (253.32 mg/g).

**Table 6:** Comparison of the Synthesized Adsorbents in the Current Study With Recent Literature

adsorbent	*C*_0_ (mg/L)	dosage (g/L)	pH	removal (%)	*Q*_m_ (mg/g)	refs
HA Removal						
magnetic chitosan	6	0.5	6	91.5	22.72	^85^
chitosan/ZIF 8	10	1.5	4	97.3	4.64	^19^
chitosan/cellulose acetate	30	0.05	4	-	184.72	^86^
Cs/AC@2:1	5	0.5	2	93	148	this study
RR120 Removal						
chitosan–epichlorohydrin biobeads	100	5	3	100	81.3	^66^
chitosan-functionalized biomass	300	1	3	99	79.35	^77^
chitosan–epichlorohydrin/zeolite	100	2	6	90.5	284.2	^67^
Cs/AC@Cur10%	100	1	3	93	143.07	this study
DCF Removal						
activated carbon–chitosan	100	1.5	6	97.02	99.29	^70^
graphene oxide–chitosan	100	1.5	5	97.06	129.26	^87^
chitosan–PEI	50	2	5	99.77	253.32	^88^
Cs/AC@Cur5%	50	1	6	97	675.01	this study

## Conclusions

The aim of this study was the synthesis of novel adsorbents for the effective removal of hazardous organic contaminants affecting wastewater quality, such as humic acid (HA), DCF, and RR120. Therefore, three chitosan/activated carbon derivatives in different molar ratios, i.e., CS/AC@1:1, CS/AC@1:2, and CS/AC@2:1, were initially synthesized and examined. Furthermore, the addition of Cur to the optimal CS/AC@2:1 derivative, resulting in two chitosan/activated carbon/curcumin derivatives, i.e., CS/AC@Cur5% and CS/AC@Cur10%, was supplementary examined, in order to analyze the possibility of additional enhancement of organic contaminants removal.

According to the results, it was assumed that the addition of Cur improved the adsorption capacity of chitosan/activated carbon derivatives in the case of DCF and RR120, but for HA removal, CS/AC@2:1 appeared more effective (93% removal at pH 2.0 with the addition of 0.5 g/L after 24 h). Hence, CS/AC@Cur5% was optimal for DCF (97% removal at pH 6.0 with the addition of 1.0 g/L after 24 h), and CS/AC@Cur10% was optimal for RR120 (94% removal at pH 3.0 with the addition of 1.0 g/L after 24 h) at 293 K. The Langmuir isotherm and PSO kinetic models were evaluated to best fit the adsorption process, proving that the adsorption of organic pollutants on CS/AC@2:1, CS/AC@Cur5%, and CS/AC@Cur10% was monolayer and matched the mechanism of chemisorption. The resulting Langmuir *Q*_*max*_ values were 109 mg/g of CS/AC2:1 for HA, 148 mg/g of CS/AC@Cur5% for DCF, and 112 mg/g of CS/AC@Cur10% for RR120.

Furthermore, a relatively simple phenomenological kinetic model was applied on a moderate basis to the numerical results and experimental data. In addition, thermodynamic analysis revealed that the adsorption was endothermic in nature (Δ*H* ^0^ > 0) and spontaneous (Δ*G* ^0^ < 0) for all three organic pollutants. The reusability of chitosan/activated carbon/curcumin nanoadsorbents for the removal of HA, DCF, and RR120 was investigated through adsorption–desorption experiments, and the relative results showed an overall reduction of approximately 15–20%, confirming that these adsorbents can be used effectively for up to six cycles of regeneration using 0.01 M NaOH solution.

Finally, full characterization is performed before and after adsorption on the optimal materials. As can be seen from SEM images, the surface of CS/AC@2:1 exhibited a highly heterogeneous and irregular structure, which, after adsorption of HA, appeared to have a rough surface with thicker pores. A similar structure with thicker pores on the surface of CS/AC@Cur5% was also observed, which was retained after DCF adsorption, again exhibiting a smooth surface. On the other hand, the adsorption of RR120 on the surface of CS/AC@Cur10% had the greatest impact, as there were some cracks in certain areas. Moreover, according to the FTIR spectra before and after adsorption, the absence of new chemical bonds was observed in all three pollutants, and a dominance of peak shifts/intensity changes, mainly shifts in the region of O–H/N–H stretching (3000–3500 cm^–1^), indicated that adsorption occurs due to surface interactions rather than covalent bonding. Overall, the application of these materials seems promising, and fixed-bed or continuous-flow studies should be conducted in the future to further evaluate their performance.

Future directions for the application of chitosan-based adsorbents for removing organic pollutants highlight their promise as sustainable, tunable, and easy-to-modify biopolymers with the potential for use at large scales in wastewater treatment. The use of nanotechnology and hybrid composites, such as chitosan-metal or magnetic chitosan matrices, can potentially allow for higher selectivity, recyclability, and multifunctionality. Nonetheless, future research must address current issues by transitioning from laboratory-scale studies to real-world applications, optimizing throughput for cost savings, and aligning with circular economy concepts to enable more sustainable remediation methods.

## Supplementary Material



## Notes

FTIR spectra of Cs/AC@2:1, Cs/AC@Cur10%, and Cs/AC@Cur5% with and without 1 h sonication (Figure S1); N_2_ isotherms of Cs/AC@2:1, Cs/AC@Cur10%, and Cs/AC@Cur5% (Figure S2) (PDF)
