# Supplementary material for: Adsorption of Dyes, Pharmaceuticals, and Humic Acids onto Chitosan Biomaterials Doped with Activated Carbon: Colloidal Approaches and Interaction Explanations
Source: Langmuir. 2025 Dec 1;41(49):33472–90. doi: 10.1021/acs.langmuir.5c05055 (PMC12713791; doi:10.1021/acs.langmuir.5c05055)
Supplement: Supplementary file 1 [file la5c05055_si_001.pdf]

Adsorption of dyes, pharmaceuticals and humic acids onto novel chitosan biomaterials doped with activated carbon: Colloidal approaches and interactions explanations

Konstantinos N. Maroulas[1], Athanasia K. Tolkou[1], Dimitrios Theologis[2], Margaritis Kostoglou[2], Ioannis A. Katsoyiannis[2], George Z. Kyzas[1,*]

1. Hephaestus Laboratory, School of Chemistry, Faculty of Sciences, Democritus University of Thrace, GR-65404 Kavala, Greece
2. Laboratory of Chemical and Environmental Technology, Department of Chemistry, Aristotle University of Thessaloniki, GR-54124, Thessaloniki, Greece

*Corresponding author(s) e-mail: kyzas@chem.duth.gr (George Z. Kyzas)

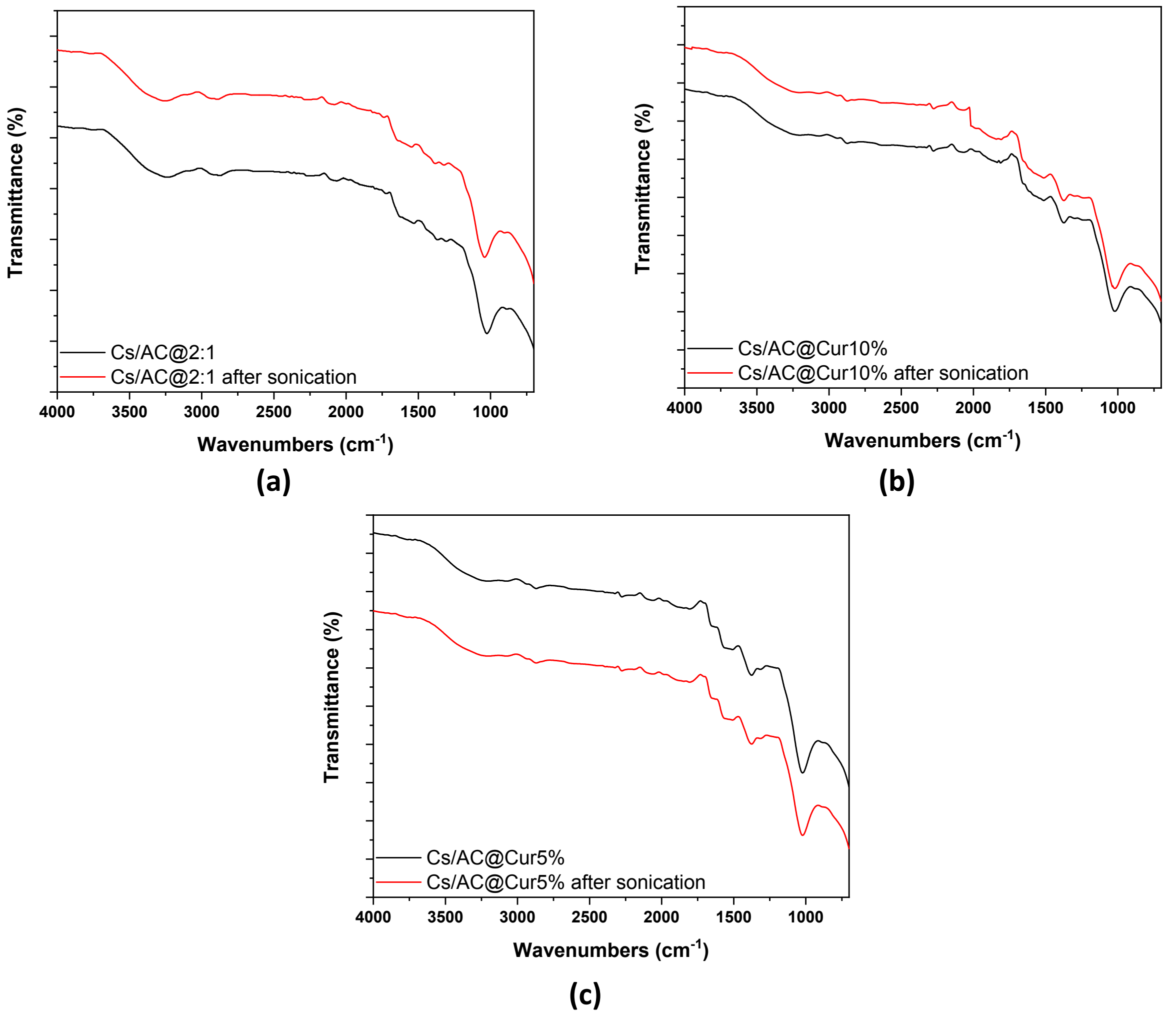

Fig. S1. FTIR spectra of **(a)** Cs/AC@2:1, **(b)** Cs/AC@Cur10% and **(c)** Cs/AC@Cur5% with and without 1h sonication

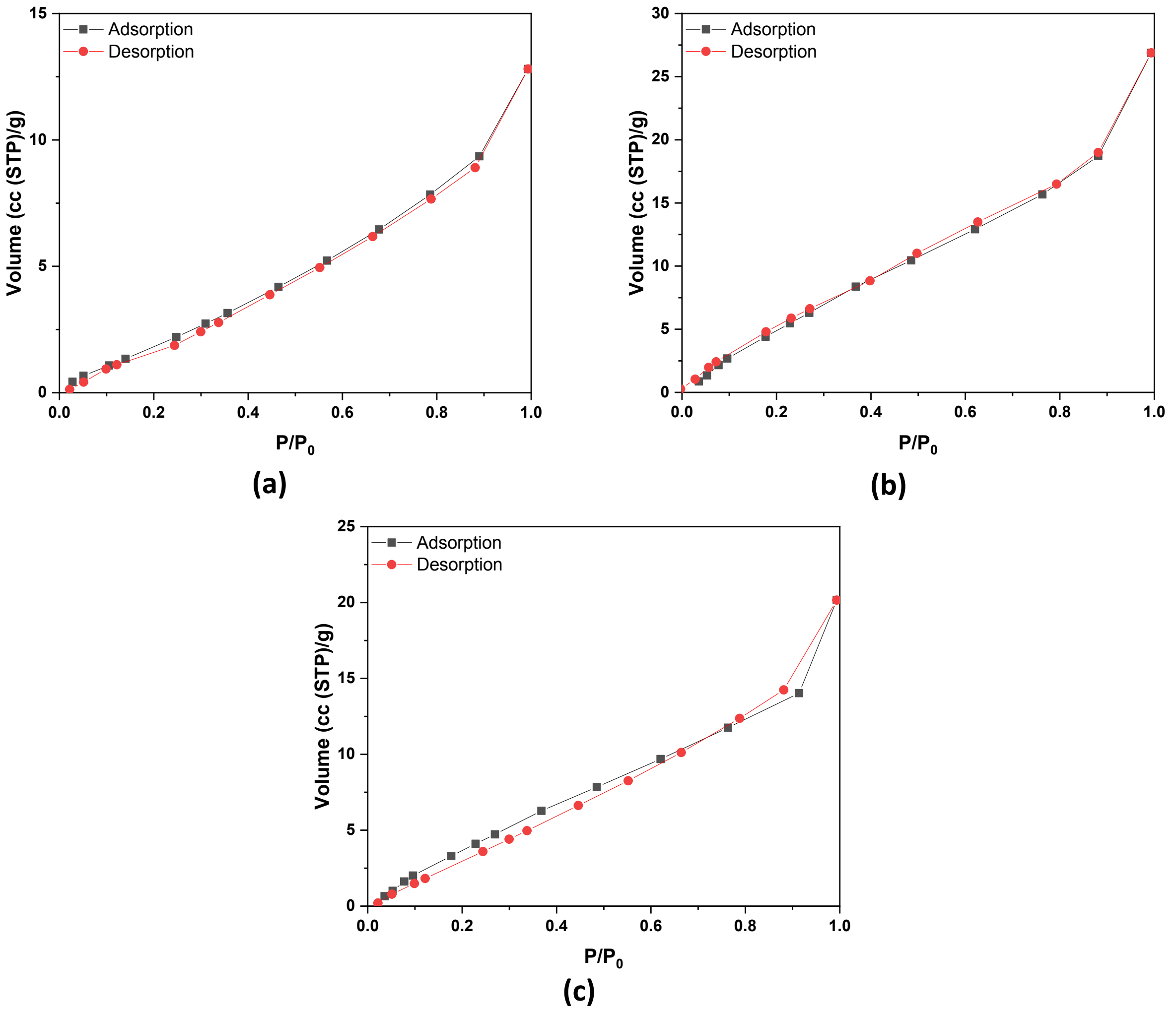

Fig. S2. N_2 isotherms of **(a)** Cs/AC@2:1, **(b)** Cs/AC@Cur10% and **(c)** Cs/AC@Cur5%
